# A Necessary and Sufficient Condition of Comparability for Using Standardized Mortality Ratio (SMR)

**DOI:** 10.2188/jea.11.24

**Published:** 2007-11-30

**Authors:** Hideto Takahashi, Masafumi Okada, Lukman Thalib, Satoshi Toyokawa, Katsumi Kano

**Affiliations:** 1 Institute of Community Medicine, University of Tsukuba.; 2 Graduate School of Medicine, University of Tsukuba.; 3 Environmental Science, Griffith University.

**Keywords:** SMR, CMF, comparability, distortion of proportionality

## Abstract

A necessary and sufficient condition of comparability for using SMR was studied mathematically by considering the equivalence between SMR and CMF, as CMF was a perfectly comparable index. This condition was expressed by either proportionality of mortality vectors or proportionality of projected person-years to the plane spanned by mortality vectors of reference and index groups. We could obtain another expression of the condition, in which affect of distortions were easily understood, which consist of three factors: distortion of proportionality of mortality, distortion of person-years and similarity of distortions.

Our results were applied to study the mortality of biliary tract cancer in Ibaraki Prefecture. Places where absolute difference between CMF and SMR exceeds some criterion (say, 0.15) were Satomi, Ushiboiri, Nihari in males and Gozenyama, Suifu and Asahi in females. All three distortion indices exceeded their upper 95% percentiles in Satomi in males.

## INTRODUCTION

One of the most frequent problems in descriptive epidemiological studies is the comparison with some measure (incidence, mortality or others) among community groups, to find high incident areas and to search for causation. If confounders like age, sex or race exist, then measures like crude rates often mislead due to the distortion of comparability. Various standardized indices have been proposed to adjust for these confounders.

Well-known indices are stratum-adjusted rates, which are weighted average with stratum-specific person-years by confounder(s). These rates are classified as a direct adjusted rate and an indirect one, or their ratio expressions, a comparative mortality figure (CMF) and a standardized mortality ratio (SMR)^1^^)^.

In this paper, CMF and SMR are studied mathematically in the context of dealing with diseases like biliary tract cancer, where case facility rates are very high. In this case mortality is also meaningful in an epidemiological sense for causation when local cancer registration is not sufficiently applicable due to imprecise registry, instability and low registry proportion.

Minimum variance properties and interval estimation have been studied for these indices^2^^)^. We find in the literature that CMF is strictly comparable but unstable with small person-years, while SMR is not satisfied for its comparability^3^^, ^^4^^)^ because of no common reference. However SMR is stable for using no stratum-adjusted estimators.

A sufficient condition of equivalence of CMF and SMR has been studied^1^^, ^^5^^, ^^6^^, ^^7^^)^ as proportionality of every stratum-specific mortality between two groups. However, the necessary and sufficient condition has not been studied and therefore the effect of distortion of proportionality is not at all clear.

This study focused on (1) a necessary and sufficient condition of equivalence, and (2) the effect of distortion of proportionality. The comparability of CMF and SMR were further studied by a real life application. We analyzed the data on mortality due to biliary tract cancer in Ibaraki Prefecture in Japan.

## DEFINITION

We denote a reference group *S*_0_, and its relevant values characterized as *i*=0, and an index group *S*_1_ and *i*=l. The group *S*_i_ consists of *J* strata and we set *j*-th stratum-specific mortality as *p_ij_* and its person-years as *n_ij_* (*i*=0,1). Then CMF and SMR are defined as,

$$CMF=\frac{\sum_{j=1}^{J}n_{0j}p_{1j}}{\sum_{j=1}^{J}n_{0j}p_{0j}},\;SMR=\frac{\sum_{j=1}^{J}n_{1j}p_{1j}}{\sum_{j=1}^{J}n_{1j}p_{0j}}.$$

If we estimate every stratum-specific rate *p_ij_* as *d_ij_*/*n_ij_* by maximum likelihood estimation with stratum-specific death number *d_ij_* being Poisson distribution, estimators of CMF and SMR are expressed as,

$$CMF=\frac{\sum_{j=1}^{J}\frac{n_{0j}}{n_{1j}}d_{1j}}{D_{0}},\;SMR=\frac{D_{1}}{\sum_{j=1}^{J}\frac{n_{1j}}{n_{0j}}d_{0j}},$$

where *D*_0_, *D*_1_ are total corresponding number of deaths.

These two indices are quite similar except that CMF is adjusted for reference person-years, while SMR is adjusted for index person-years. Additionally, they can be recognized weighted means of stratum-specific mortalities with weights being each person-years, which are also expressed by inner products in the *J*-dimensional Euclidean space:

$$CMF=\frac{\left(n_{0},p_{1}\right)}{\left(n_{0},p_{0}\right)},\;SMR=\frac{\left(n_{1},p_{1}\right)}{\left(n_{1},p_{0}\right)},$$

with *n_i_*= (*n_i_*_1_, ···, *n_iJ_*)′ and *p_i_*= (*p_i_*_1_, ···, *p_iJ_*)′. The prime ′ denotes transpose notation.

In general inner product (a,b) with two *J*×1 vectors of *a*= (*a*_1_,···, *a_J_*)′, *b*= (*b*_1_,···, *b_J_*)′ is defined by (a,b)= ||a||||b||cos *θ*, which equals to $\sum_{j=1}^{J}a_{j}b_{j}$ , with $||\text{a}||=\sqrt{\sum_{j=1}^{J}a_{j}^{2}}$ and *θ* being an angle (0° ≦ *θ* ≦ 180°) between two vectors *a*,*b*.

## A NECESSARY AND SUFFICIENT CONDITION OF EQUIVALENCE OF SMR AND CMF

We suppose *π* as a plane spanned by two vectors *p*_0_, *p*_1_ and projected vectors of *n*_0_, *n*_1_ to *π* as $n_{0}^{\left(0\right)}=(n_{01}^{\left(0\right)},\ldots ,n_{0J}^{\left(0\right)}{)}^{\prime }$ , $n_{1}^{\left(0\right)}=(n_{11}^{\left(0\right)},\ldots ,n_{1J}^{\left(0\right)}{)}^{\prime }$ . As $n_{0}-n_{0}^{\left(0\right)}$ , $n_{1}-n_{1}^{\left(0\right)}$ are orthogonal to *π*, then CMF and SMR can be expressed as

$$SMR=\frac{\left(n_{1}^{\left(0\right)},p_{1}\right)}{\left(n_{1}^{\left(0\right)},p_{0}\right)},\;CMF=\frac{\left(n_{0}^{\left(0\right)},p_{1}\right)}{\left(n_{0}^{\left(0\right)},p_{0}\right)}.$$

Now we can obtain the next necessary and sufficient condition of comparability based the equivalence of SMR and CMF using above expression.

[A necessary and sufficient condition of comparability for using SMR]

*A necessary and sufficient condition of CMF=SMR is $n_{1}^{\left(0\right)}=kn_{0}^{\left(0\right)}$ or $p_{1}=lp_{0}$ *,* (with k*,* l being constants)*.

[Proof] We can also obtain the next equivalent expression of SMR-CMF.

$$\text{SMR–CMF}=\frac{1}{\left(n_{1}^{\left(0\right)},p_{0}\right)}\sum_{j=1}^{J}\left(p_{1j}-\frac{\left(n_{0}^{\left(0\right)},p_{1}\right)}{\left(n_{0}^{\left(0\right)},p_{0}\right)}p_{0j}\right)\left(n_{1j}^{\left(0\right)}-\frac{\left(n_{1}^{\left(0\right)},p_{0}\right)}{\left(n_{0}^{\left(0\right)},p_{0}\right)}n_{0j}^{\left(0\right)}\right)$$ (1)

$$=\frac{1}{\left(n_{1}^{\left(0\right)},p_{0}\right)}\|\eta \|\|\tau \|\cos\psi$$ (2)

where $\eta =(p_{1}-(n_{0}^{\left(0\right)},p_{1})/(n_{0}^{\left(0\right)},p_{0})p_{0})$ , $\tau =(n_{1}^{\left(0\right)}-(n_{1}^{\left(0\right)},p_{0})/(n_{0}^{\left(0\right)},p_{0})n_{0}^{\left(0\right)})$ , and *ψ* to be an angle of *η* and *τ*, (0° ≦ *ψ* ≦ 180°).

Thus we can obtain that SMR=CMF is equivalent to || *τ* ||= 0 or || *η* ||= 0 or *ψ* = 90°. Here || *τ* ||=0 ⇔ $n_{1}^{\left(0\right)}=kn_{0}^{\left(0\right)}$ and || *η* ||=0 ⇔ *p*_1_=*lp*_0_. However $\eta ⊥n_{0}^{\left(0\right)}$ , *τ* ⊥ *p*_0_ and *η*, *τ*, *p*_0_, $n_{0}^{\left(0\right)}$ spans the same plane *π*. $\sum_{i=}^{J}=D_{0}\neq 0$ . Therefore ( *η*, *τ* ) ≠ 0 if *η* ≠ 0 and *τ* ≠ 0, which is *ψ* ≠ 90°. It completes the proof.

## EFFECT OF DISTORTION OF PROPORTIONALITY

In the previous section, we could obtain a necessary and sufficient condition of equivalence of SMR and CMF, as proportionality of projected person-years or proportionality of mortalities. In this section, we describe the effect of distortion of proportionality with n_0_, n_1_ instead of $n_{0}^{\left(0\right)}$ , $n_{1}^{\left(0\right)}$ .

From the expression (1), we can consider that *η* = (*p*_1_ - (*n*_0_, *p*_1_) / (*n*_0_, *p*_0_) *p*_0_) would express distortion of proportionality of mortalities and *τ* = (*n*_1_ - (*n*_1_, *p*_0_) / (*n*_0_, *p*_0_) *n*_0_*_j_*) would express distortion of proportionality of person-years. Then effects of distortion of proportionality can be expressed by (2), where || *η* || is a level of distortion of mortalities, and || *τ* || is that of person-years and |cos *ψ*| would be a level of similarity of both distortions.

## A REAL LIFE APPLICATION

Using the data set available on average mortality of biliary tract cancer (ICD9= 156.0,156.1, 156.8, 156.9) in Ibaraki Prefecture (1992-1997), the comparability of SMR and CMF were analyzed in Table 1 in males and Table 2 in females. Cities and villages where absolute differences (i.e. SMR-CMF) exceeded 0.15 are Satomi (-0.878), Ushibori (0.196) and Niihari (-0.231) in males and Gozenyama (-0.178), Suifu (-0.164) and Asahi (-0.234) in females. If we set the criterion of difference as upper 5% percentiles of || *η* ||, || *τ* || and |cos *ψ|*, then they become 54.7, 0.233 and 0.116 for males and 30.5, 0.222 and 0.143 for females, respectively.

**Table 1.** SMRs and CMFs of Bilirary Tract Cancer in Ibaraki Prefecture (Males).

Places	Person-years (1996)	Averaged death number	SMR	CMF	Difference	|| *η* ||	|| *τ* ||	|cos *ψ*|
Mito	120,183	10.0	0.858	0.855	0.003	4.41	0.015	0.048
Hitachi	100,537	10.2	1.013	1.024	-0.011	4.63	0.036	0.068
Tsuchiura	66,084	7.0	1.140	1.132	0.008	2.07	0.027	0.135
Koga	29,262	2.7	0.877	0.883	-0.006	6.71	0.021	0.045
Ishioka	25,916	3.7	1.409	1.412	-0.003	13.74	0.013	0.018
Shimodate	33,071	2.8	0.850	0.852	-0.002	9.05	0.008	0.027
Yuki	26,776	4.0	1.449	1.458	-0.009	8.92	0.016	0.066
Ryugasaki	35,557	2.0	0.682	0.684	-0.001	8.97	0.062	0.002
Shimotsuma	18,216	0.3	0.169	0.177	-0.008	4.20	0.031	0.069
Mitsukaido	21,155	1.8	0.743	0.746	-0.003	15.10	0.048	0.005
Hitachi-ota	19,291	2.7	1.026	0.934	0.092	18.53	0.105	0.064
Takahagi	17,564	1.7	0.848	0.857	-0.009	14.25	0.042	0.018
Kitaibaraki	25,806	2.2	0.714	0.723	-0.010	10.88	0.060	0.017
Kasama	14,717	2.3	1.267	1.253	0.013	18.78	0.076	0.012
Toride	42,105	2.7	0.810	0.791	0.020	12.21	0.077	0.017
Iwai	22,306	1.8	0.808	0.801	0.006	10.88	0.012	0.051
Ushiku	33,060	2.7	1.024	0.979	0.045	9.42	0.076	0.049
Tsukuba	81,453	6.3	1.004	0.999	0.005	11.20	0.092	0.004
Hitachinaka	73,956	5.5	0.879	0.855	0.024	8.26	0.052	0.048
Kashima	31,399	3.2	1.307	1.405	-0.098	38.28	0.072	0.027
Ibaraki	17,744	1.8	0.878	0.874	0.004	15.32	0.058	0.005
Ogawa	10,154	0.7	0.669	0.661	0.008	12.65	0.044	0.014
Minori	11,907	1.7	1.350	1.357	-0.007	24.48	0.019	0.015
Uchihara	7,459	0.7	0.771	0.811	-0.040	14.06	0.051	0.065
Johoku	6,099	0.7	0.864	0.866	-0.002	14.21	0.085	0.002
Katsura	3,401	0.5	0.901	0.796	0.105	23.96	0.183	0.039
Gozenyama	2,316	0.2	0.431	0.377	0.055	12.73	0.198	0.036
Oarai	10,062	0.5	0.408	0.374	0.033	9.64	0.067	0.063
Tomobe	16,690	1.0	0.628	0.607	0.021	13.62	0.020	0.071
Iwama	8,264	0.8	0.868	0.808	0.060	19.20	0.052	0.070
Nanakai	1,295	0.5	2.631	2.705	-0.074	68.39	0.157	0.010
Iwase	11,381	1.3	0.973	1.038	-0.065	11.15	0.064	0.109
Tokai	16,548	1.7	1.119	1.145	-0.026	18.92	0.048	0.026
Naka	22,113	2.3	0.934	0.918	0.015	10.68	0.043	0.038
Urizura	4,387	1.0	1.728	1.693	0.035	28.89	0.102	0.016
Omiya	12,953	1.2	0.690	0.642	0.048	13.33	0.092	0.052
Yamagata	4,161	0.8	1.099	1.057	0.042	20.85	0.239	0.015
Miwa	2,429	0.2	0.385	0.304	0.081	10.27	0.233	0.061
Ogawa	2,333	0.8	1.879	1.874	0.005	71.84	0.267	0.001
Kanasago	5,218	0.5	0.555	0.501	0.053	32.62	0.215	0.013
Suifu	3,304	0.3	0.515	0.475	0.041	11.35	0.281	0.025
Satomi	2,238	0.7	1.592	2.470	-0.878	54.74	0.260	0.116
Daigo	12,476	2.7	1.230	1.183	0.047	25.59	0.222	0.014
Juoh	6,325	0.7	0.976	0.871	0.105	24.30	0.044	0.106
Asahi	5,623	0.7	0.948	1.011	-0.064	26.96	0.081	0.037
Hokota	14,121	2.2	1.252	1.326	-0.074	13.37	0.074	0.093
Taiyo	5,454	1.5	1.953	2.177	-0.225	34.56	0.127	0.072
Kamisu	23,329	2.8	1.889	2.000	-0.111	21.14	0.108	0.031
Hasaki	19,594	3.7	2.223	2.220	0.003	15.77	0.053	0.003
Aso	8,531	1.3	1.145	1.167	-0.022	9.10	0.113	0.030
Ushibori	3,091	1.3	3.553	3.358	0.196	57.96	0.073	0.056
Itako	12,721	2.8	2.405	2.547	-0.141	30.52	0.032	0.134
Kitaura	5,422	1.0	1.371	1.472	-0.101	24.13	0.111	0.051
Tamatsukuri	6,996	1.3	1.497	1.404	0.093	8.85	0.090	0.150
Edosaki	10,039	0.2	0.169	0.177	-0.008	8.35	0.032	0.029
Miho	9,052	0.5	0.608	0.610	-0.003	13.20	0.035	0.005
Ami	22,898	2.3	1.186	1.175	0.012	18.29	0.045	0.012
Kukizaki	13,022	0.7	0.617	0.505	0.112	17.27	0.089	0.061
Shintone	5,278	0.2	0.265	0.223	0.043	14.50	0.067	0.052
Kawachi	5,715	0.5	0.694	0.672	0.022	21.41	0.080	0.016
Sakuragawa	3,871	1.2	2.262	2.306	-0.044	62.58	0.106	0.009
Azuma	6,483	1.5	1.717	1.666	0.051	32.88	0.110	0.019
Dejima	9,513	1.3	1.000	0.952	0.047	22.90	0.116	0.025
Tamari	4,422	0.3	0.703	0.746	-0.043	24.00	0.033	0.058
Yasato	15,186	1.3	0.647	0.658	-0.010	14.61	0.109	0.009
Chiyoda	13,554	1.8	1.758	1.740	0.018	16.58	0.073	0.011
Niihari	4,743	1.2	1.807	2.038	-0.231	30.27	0.105	0.098
Ina	13,007	0.8	0.688	0.556	0.132	12.41	0.052	0.189
Yawara	7,132	0.2	0.221	0.232	-0.011	7.84	0.024	0.060
Sekijo	8,106	0.2	0.179	0.176	0.003	8.31	0.048	0.007
Akeno	9,143	0.5	0.467	0.462	0.006	16.38	0.052	0.008
Makabe	10,187	0.8	0.666	0.635	0.031	14.85	0.071	0.036
Yamato	3,854	0.7	1.408	1.405	0.003	30.28	0.077	0.002
Kyowa	8,570	1.2	1.122	1.099	0.023	25.75	0.062	0.017
Yachiyo	12,536	1.3	0.925	0.876	0.049	9.42	0.052	0.116
Chiyokawa	4,692	0.2	0.316	0.287	0.029	18.64	0.040	0.044
Ishige	11,745	2.0	1.637	1.623	0.014	19.32	0.019	0.041
Sowa	24,256	1.5	0.852	0.888	-0.036	7.75	0.084	0.040
Goka	5,212	0.2	0.309	0.287	0.022	9.70	0.037	0.062
Sanwa	19,993	2.0	1.267	1.245	0.021	8.51	0.067	0.029
Sashima	7,757	1.2	1.316	1.311	0.005	32.95	0.054	0.003
Sakai	13,612	2.2	1.614	1.601	0.013	29.46	0.015	0.029
Moriya	23,195	1.3	0.875	0.913	-0.038	10.73	0.111	0.021
Fujishiro	16,509	0.5	0.357	0.392	-0.035	13.94	0.067	0.031
Tone	9,830	1.2	1.327	1.324	0.003	35.54	0.088	0.001

		mean	1.061	1.067	-0.006	19.34	0.081	0.042
		SD	0.594	0.625	0.115	13.79	0.062	0.038
		CV	0.560	0.586	-18.594	0.71	0.764	90.300

		95%percentile(U)				54.70	0.233	0.116

**Table 2.** SMRs and CMFs of Bilirary Tract Cancer in Ibaraki Prefecture (Females).

Places	Person-years (1996)	Averaged death number	SMR	CMF	Difference	|| *η* ||	|| *τ* ||	|cos *ψ*|
Mito	125,477	12.8	0.821	0.821	-0.001	2.92	0.026	0.013
Hitachi	98,600	19.5	1.467	1.476	-0.009	8.01	0.027	0.055
Tsuchiura	66,397	6.5	0.808	0.802	0.006	5.05	0.034	0.042
Koga	29,866	3.5	0.905	0.938	-0.033	11.39	0.023	0.166
Ishioka	26,707	3.2	0.874	0.871	0.003	8.96	0.014	0.031
Shimodate	32,967	6.2	1.378	1.375	0.003	6.39	0.007	0.100
Yuki	27,071	3.2	0.855	0.855	0.000	7.25	0.014	0.006
Ryugasaki	34,321	3.2	0.817	0.812	0.005	7.80	0.050	0.014
Shimotsuma	17,979	2.0	0.746	0.760	-0.013	6.66	0.038	0.079
Mitsukaido	21,595	3.8	1.093	1.093	0.000	10.33	0.059	0.000
Hitachi-ota	20,372	3.3	0.939	0.891	0.049	7.45	0.088	0.129
Takahagi	18,043	2.0	0.732	0.748	-0.016	4.93	0.042	0.120
Kitaibaraki	26,372	2.7	0.663	0.640	0.023	5.92	0.049	0.119
Kasama	15,615	1.7	0.613	0.601	0.011	12.75	0.088	0.018
Toride	42,509	4.8	1.127	1.191	-0.065	25.11	0.088	0.030
Iwai	21,805	3.3	1.106	1.097	0.009	15.35	0.011	0.072
Ushiku	33,617	2.2	0.656	0.707	-0.050	11.41	0.092	0.047
Tsukuba	75,146	8.7	1.042	1.043	-0.001	10.97	0.070	0.002
Hitachinaka	72,754	8.8	1.107	1.089	0.018	8.16	0.057	0.043
Kashima	29,279	3.0	0.909	0.913	-0.004	13.83	0.052	0.006
Ibaraki	17,927	1.8	0.658	0.664	-0.006	7.46	0.050	0.025
Ogawa	9,418	0.8	0.620	0.603	0.017	7.34	0.035	0.094
Minori	12,053	1.2	0.726	0.710	0.016	18.03	0.008	0.154
Uchihara	7,470	0.8	0.719	0.757	-0.038	16.73	0.045	0.078
Johoku	6,338	1.7	1.477	1.407	0.070	15.71	0.098	0.080
Katsura	3,550	0.3	0.449	0.390	0.059	27.56	0.166	0.027
Gozenyama	2,425	0.5	0.898	1.077	-0.178	20.23	0.209	0.097
Oarai	10,357	1.5	0.883	0.920	-0.036	8.89	0.071	0.095
Tomobe	17,347	2.0	0.901	0.899	0.002	12.53	0.019	0.013
Iwama	8,348	1.0	0.833	0.877	-0.045	9.88	0.031	0.214
Nanakai	1,315	0.7	2.329	2.381	-0.051	85.33	0.189	0.007
Iwase	12,074	1.8	0.865	0.878	-0.013	11.65	0.091	0.022
Tokai	16,230	2.7	1.435	1.449	-0.014	20.75	0.053	0.014
Naka	23,024	1.8	0.576	0.583	-0.007	10.67	0.014	0.065
Urizura	4,753	0.7	0.812	0.664	0.148	29.54	0.089	0.097
Omiya	13,534	2.2	0.959	0.941	0.018	9.43	0.073	0.044
Yamagata	4,359	0.8	0.812	0.790	0.022	22.82	0.222	0.010
Miwa	2,523	0.2	0.280	0.299	-0.019	10.14	0.228	0.019
Ogawa	2,522	0.7	1.112	1.013	0.099	33.08	0.233	0.031
Kanasago	5,540	1.0	0.787	0.720	0.067	8.22	0.209	0.090
Suifu	3,428	1.5	1.740	1.904	-0.164	23.99	0.259	0.066
Satomi	2,299	0.0	0.000	0.000	0.000	0.00	0.226	0.000
Daigo	13,088	2.0	0.675	0.675	0.001	9.59	0.209	0.001
Juoh	6,684	2.0	2.127	2.123	0.003	26.79	0.029	0.006
Asahi	5,823	1.2	1.224	1.458	-0.234	18.44	0.071	0.291
Hokota	14,519	2.8	1.231	1.223	0.008	10.14	0.060	0.021
Taiyo	5,442	0.8	0.856	0.859	-0.003	9.91	0.110	0.005
Kamisu	21,478	1.8	0.906	0.896	0.010	11.13	0.090	0.010
Hasaki	19,214	2.3	1.002	0.998	0.004	6.22	0.038	0.018
Aso	8,710	2.3	1.456	1.548	-0.092	17.56	0.113	0.085
Ushibori	3,118	2.3	4.358	4.248	0.109	50.22	0.087	0.043
Itako	13,219	2.8	1.707	1.677	0.030	30.52	0.024	0.053
Kitaura	5,484	1.3	1.370	1.225	0.145	26.00	0.109	0.091
Tamatsukuri	7,164	2.8	2.273	2.344	-0.071	16.21	0.095	0.080
Edosaki	10,109	1.2	0.935	1.022	-0.087	26.88	0.034	0.118
Miho	8,780	0.5	0.431	0.435	-0.004	8.09	0.026	0.026
Ami	22,848	3.2	1.251	1.273	-0.022	18.71	0.054	0.024
Kukizaki	13,365	0.8	0.646	0.686	-0.040	15.54	0.110	0.023
Shintone	5,265	1.5	1.703	1.647	0.056	26.99	0.077	0.045
Kawachi	5,990	0.7	0.629	0.584	0.045	13.01	0.098	0.063
Sakuragawa	3,980	1.0	1.239	1.314	-0.075	27.64	0.151	0.037
Azuma	6,745	2.2	1.646	1.594	0.052	12.24	0.138	0.060
Dejima	9,570	2.2	1.235	1.257	-0.022	28.53	0.105	0.013
Tamari	4,303	1.2	1.893	1.906	-0.013	19.18	0.024	0.040
Yasato	15,554	3.2	1.133	1.198	-0.065	12.60	0.103	0.090
Chiyoda	12,712	0.5	0.385	0.431	-0.046	18.59	0.072	0.036
Niihari	4,849	0.7	0.747	0.751	-0.004	15.46	0.105	0.005
Ina	13,190	1.5	0.917	0.877	0.040	7.79	0.053	0.119
Yawara	7,186	1.5	1.479	1.538	-0.058	33.64	0.024	0.102
Sekijo	8,315	1.0	0.770	0.762	0.008	10.37	0.050	0.023
Akeno	9,098	1.2	0.788	0.742	0.046	16.96	0.063	0.070
Makabe	10,480	1.7	0.936	0.917	0.018	9.04	0.081	0.042
Yamato	3,915	0.5	0.724	0.866	-0.143	17.97	0.098	0.143
Kyowa	8,819	1.8	1.255	1.297	-0.042	17.40	0.067	0.060
Yachiyo	12,496	2.7	1.372	1.399	-0.028	15.04	0.056	0.051
Chiyokawa	4,696	0.7	0.874	0.849	0.024	19.95	0.062	0.032
Ishige	11,689	1.7	1.019	1.019	0.000	17.98	0.018	0.002
Sowa	22,923	2.5	1.056	1.037	0.019	6.63	0.071	0.042
Goka	5,091	0.3	0.484	0.471	0.013	18.80	0.031	0.030
Sanwa	19,929	2.0	1.001	1.026	-0.026	11.12	0.078	0.030
Sashima	7,628	1.5	1.227	1.164	0.063	11.91	0.062	0.138
Sakai	13,639	2.2	1.152	1.136	0.016	27.00	0.017	0.050
Moriya	22,773	1.0	0.486	0.494	-0.008	10.26	0.108	0.006
Fujishiro	17,253	1.2	0.559	0.573	-0.014	4.77	0.058	0.062
Tone	10,320	1.0	0.836	0.859	-0.023	9.53	0.090	0.031

		mean	1.041	1.048	-0.006	15.80	0.080	0.056
		SD	0.556	0.555	0.059	11.43	0.1	0.1
		CV	0.534	0.530	-9.463	0.72	0.746	91.633

		95%percentile(U)				30.50	0.222	0.143

The values of || *η* ||, || *τ* ||, |cos *ψ*| for above places based on data in males were 54.7, 0.260, 0.116 for Satomi, 58.0, 0.073, 0.056 for Ushibori, 30.3, 0.105, 0.098 for Niihari, and in females were 20.2, 0.209, 0.097 for Gozenyama, 24.0, 0.259, 0.066 for Suifu, 18.4, 0.071, 0.291 for Asahi.

From this, we found in males that Satomi was caused by all three values || *η* ||, || *τ* |, |cos *ψ*| Ushibori was caused by mainly || *η* ||. Males of Niihari were not clear but considered by comparatively high values of three values. In females, Suifu Vil. were caused by mainly || *τ* ||, Asahi Vil. were by mainly |cos *ψ*|. Satomi was not clear but considered by comparatively high values of three values.

A necessary and sufficient condition of equivalence of SMR and CMF was found in this paper to be proportional to stratum-specific projected person-years to the plane spanned by *p*_0_, *p*_1_ or proportionality of stratum-specific mortalities. || *η* ||, || *τ* ||, |cos *ψ*| indicates the difference between SMR and CMF. Especially, || *η* || represents a distortion of proportion of stratum-specific mortalities, and || *τ* || represents a distortion of proportion of stratum person-years, while |cos *ψ*| is a similarity of both distortions, || *η* || and || *τ* ||.

The parts which distort comparability could be found in the components of *η*, *τ* in a real life application. For example, for males of Satomi the expected death number was 0.419 versus the actual death number of 0.667 (SMR=1.59), and adjusted in the reference (Ibaraki Prefecture), while the expected total death number in Satomi was 366 and the actual death number was 148 (CMF=2.47). Studying the difference between SMR and CMF where all || *η* || =54.7, *τ* =0.260, |cos *ψ*|= 0.115 exceeded the criterion, causation of this difference was made clear by the search of *p*_1_*_j_*- (*n*_0_, *p*_1_) / (*n*_0_, *p*_0_)*p*_0_*_j_* and *n*_1_*_j_*-(*n*_1_, *p*_0_) / (*n*_0_, *p*_0_)*n*_0_*_j_*. In Satomi, we found that this difference was caused by four categories over 70 years old and had 0 death numbers and low proportion of person-years in 20-24 year-old category.

From estimation point of view, the smaller expected death number gives instability of mortality caused by large random variation of estimators. Even when we have to avoid this by reclassifying, 0-39, 40-64, 65- for instance, our results of difference did not change largely. In this case, we obtained SMR= 1.65 and CMF= 2.97 in Satomi. Ultimately, we can consider adopting only one stratum and use the comparison conducted by crude rate. This method can achieve to maximize the death number for each stratum, which is reasonable by precision of estimators. However an original objective to adjust effects influenced by confounders cannot be attained. Number of strata used by adjustment is controversial under the estimation procedure. If some estimation procedure recommended to small number of categories, results would cause distortion of comparability due to confounders even if they were reasonable to estimate. In these cases, comparison with SMRs would be of no use. in the strict sense.

From stability point of view, empirical Bayes method has been developed and applied to estimate SMRs^8^^, ^^9^^)^. This method requires an assumption of the prior distribution being normal, gamma distribution or some other reasonable distribution, and estimators of SMR are given by expectation of posterior distribution. When we applied gamma prior distribution, it is mathematically easy to handle, and it results reasonably that estimated mortalities of index groups (cities, villages) with small size (person-years) tend to move towards overall mean. Therefore selection of gamma prior is convenient for epidemiologists. However there are many prior distributions, care of understanding is necessary as prior distribution often tends to be selected by mathematical request more than by epidemiological consideration.

Equivalent transformation of SMR-CMF gave a necessary and sufficient condition to be proportionality of stratum-specific mortalities or of projected person-years, which included proportionalities of stratum-specific mortalities or of person-years to be intuitively understood. To obtain the necessary condition, consideration on the adjusted solution space was necessary. As distortion of proportionality was expressed by || *η* ||, || *τ* ||, |cos *ψ*|, we could specify the strata in which contained the large distortion in the three points: mortality, person-years and their similarities. From estimation framework, we must consider not only comparability but also stability of estimators, with combination of some strata to increase expected death number, for example. This problem relates to necessity of adjustment may need further study and discussion.
